# Hydroxypropyltrimethyl Ammonium Chloride Chitosan Functionalized-PLGA Electrospun Fibrous Membranes as Antibacterial Wound Dressing: In Vitro and In Vivo Evaluation

**DOI:** 10.3390/polym9120697

**Published:** 2017-12-11

**Authors:** Shengbing Yang, Xiuguo Han, Yuhang Jia, Hongbo Zhang, Tingting Tang

**Affiliations:** 1 Shanghai Key Laboratory of Orthopaedic Implants, Department of Orthopaedic Surgery, Shanghai Ninth People’s Hospital, Shanghai Jiao Tong University School of Medicine, Shanghai 200011, China; bioshengbingy@163.com (S.Y.); doctorhanxiuguo@163.com (X.H.); 2 School of Mechanical and Power Engineering, East China University of Science and Technology, Shanghai 200237, China; jyhdds@163.com

**Keywords:** electrospun nanofibers, hydroxypropyltrimethyl ammonium chloride chitosan (HACC), antibacterial activity, functional nanofibers, wound dressing

## Abstract

A novel poly(lactic-*co*-glycolic acid) (PLGA)-hydroxypropyltrimethyl ammonium chloride chitosan (HACC) composite nanofiber wound dressing was prepared through electrospinning and the entrapment-graft technique as an antibacterial dressing for cutaneous wound healing. HACC with 30% degrees of substitution (DS) was immobilized onto the surface of PLGA membranes via the reaction between carboxyl groups in PLGA after alkali treatment and the reactive groups (–NH_2_) in HACC molecules. The naked PLGA and chitosan graft PLGA (PLGA-CS) membranes served as controls. The surface immobilization was characterized by scanning electron microscopy (SEM), atomic force microscopy (AFM), Fourier transform infrared (FTIR), thermogravimetric analysis (TGA) and energy dispersive X-ray spectrometry (EDX). The morphology studies showed that the membranes remain uniform after the immobilization process. The effects of the surface modification by HACC and CS on the biological properties of the membranes were also investigated. Compared with PLGA and PLGA-CS, PLGA-HACC exhibited more effective antibacterial activity towards both Gram-positive (*S. aureus*) and Gram-negative (*P. aeruginosa*) bacteria. The newly developed fibrous membranes were evaluated in vitro for their cytotoxicity using human dermal fibroblasts (HDFs) and human keratinocytes (HaCaTs) and in vivo using a wound healing mice model. It was revealed that PLGA-HACC fibrous membranes exhibited favorable cytocompatibility and significantly stimulated adhesion, spreading and proliferation of HDFs and HaCaTs. PLGA-HACC exhibited excellent wound healing efficacy, which was confirmed using a full thickness excision wound model in *S. aureus*-infected mice. The experimental results in this work suggest that PLGA-HACC is a strong candidate for use as a therapeutic biomaterial in the treatment of infected wounds.

## 1. Introduction

The healing of wounds, especially extensive full-thickness wounds, is one of the most challenging clinical problems [1,2]. Skin wound dressing is of significant importance in wound healing, as it prevents bacterial contamination, absorbs excess exudates, ensures sufficient gas and nutrient exchange and maintains a moist environment for cell proliferation and migration [3,4,5]. A range of dressing types has been fabricated and used in accelerating wound healing and skin regeneration.

Various processing techniques, such as lyophilization, 3D printing, solvent casting and electrospinning, can be used to produce membranes for wound dressing [6,7,8,9,10]. Electrospun nanofibrous membranes display high porosity with excellent pore interconnectivity and exhibit unique advantages for functional wound dressing materials [11,12,13]. The nanofibrous membranes can provide an effective physical barrier to protect the open wound from further physical damage and contamination from exogenous micropathogens and serve as a template for the skin cells in the self-repairing process [14]. To date, various polymers, ranging from natural to synthetic polymers or hybrid blends of both, have been electrospun into nanofibers, and these fibers have been widely used as wound dressing [15,16]. Bacterial infection is one of major problems in wound care, because infection can cause the formation of exudate, induce an inflammatory reaction, prolong the treatment time and even endanger life [17,18,19]. Microbes can enter the body through open wounds and go in to deeper portions of the tissue [20,21]. Various biocidal substances like silver, antibiotics, chitosan, iodine and poly-ammonium salts have been incorporated into nanofibrous membranes to produce an antibacterial effect [22,23,24,25]. However, the adverse effects and systemic toxicity of these antibacterial materials restrict their effectiveness [26,27]. Anti-bacterial wound dressings have become increasingly popular in recent years, with most commercial suppliers now offering silver-coated or nanoparticle-impregnated dressings [28,29,30]. However, several studies have also reported the cytotoxicity of silver to mammalian cells and the negative impact of silver on the wound healing process [31,32]. Therefore, there is an apparent need for more effective antimicrobial agents suitable for use in both preventative measures and the prevention of microbial colonization in open wounds; these agents must be further investigated. Our group has prepared hydroxypropyltrimethyl ammonium chloride chitosan (HACC), a chitosan derivative, which has excellent biocompatibility and adjustable antibacterial activity [33]. Furthermore, the prominent antibacterial activity of HACC, either when loaded in bone cement or when serving as a coating material, was demonstrated both in vitro and in vivo [34,35,36,37,38]. 

Poly(lactic-*co*-glycolic acid) (PLGA) is considered one of the most successfully synthesized biodegradable polymers for its good biocompatibility, adjustable mechanical properties and tunable degradation rate [39]. In this study, a composite of HACC-*grafted*-PLGA nanofibers (PLGA-HACC) was prepared via an electrospinning technique and surface modification. The study was designed to compare PLGA-HACC with chitosan-grafted PLGA membranes (PLGA-CS) and PLGA-only membranes by evaluating their antibacterial potential against *Staphylococcus aureus* (ATCC25923) and *Pseudomonas aeruginosa* (ATCC 9027). Their cytocompatibility with both human dermal fibroblast cells (HDFs) and keratinocyte cells (HaCaTs) was also evaluated. Finally, an in vivo *S. aureus*-infected wound model with full-thickness skin defects was used to confirm the ability of PLGA-HACC fibrous membranes to accelerate the repairing of infected skin.

## 2. Materials and Methods

### 2.1. Materials

Poly(d,l-lactide-*co*-glycolide) (PLGA, *M*_W_ ≈ 200,000) with a –COOH end-group was purchased from the (Shandong Institute of Medical Instruments, Jinan, China). Chitosan (*M*_W_ ≈ 200 kDa, with a deacetylation degree (DD) of 95%) was purchased from the Biochemical Medicine Plant of Qingdao (Qingdao, China). *N*-hydroxysuccinimide (NHS), hexafluoroisopropanol (HFIP), 1-(3-dimethylaminopropyl)-3-ethylcarbodiimide hydrochloride (EDC·HCl) and 2-morpholinoethane sulfonic acid (MES) were purchased from Sigma Aldrich (St. Louis, MO, USA). *Staphylococcus aureus* (ATCC 25923) and *Pseudomonas aeruginosa* (ATCC 9027) were maintained in our laboratory. Human dermal fibroblast cells (HDFs) and keratinocyte cells (HaCaTs) were purchased from the (Kunming Animal Institute, Kunming, China).

### 2.2. Preparation of PLGA Nanofibrous Membranes

The PLGA nanofiber membranes were electrospun as previously reported [12]. Firstly, a 10 wt % PLGA solution was prepared by dissolving the polymer with HFIP. The polymer solution was then fed into a syringe capped with an internal diameter of 0.35 mm. A DC voltage of 12 kV potential was applied between the syringe tip and an aluminum sheet-collector at a distance of 15–20 cm and at a syringe flow rate of 1.5–2.0 mL/h at an ambient temperature of 25 °C. The fibers were then dried in a vacuum oven at 37 °C for 48 h to remove residual HFIP. 

### 2.3. Preparation of HACC, CS-Conjugated PLGA Nanofibrous Membranes

HACC with 30% degrees of substitution (DS) was prepared according to a previously described modified method [33]. The prepared PLGA nanofibrous membranes were soaked in NaOH aqueous solution (50 mg·mL^−1^) for 2 h to form reactive carboxyl groups followed by a thorough rinse using abundant distilled water. Subsequently, the PLGA nanofibrous membranes were cross-linked with 0.2% HACC in EDC (0.40 g)/NHS (0.097 g)/MES (50 mL) solution for 24 h. Finally, the PLGA-HACC fibrous membrane was rinsed with abundant distilled water and then dried. Chitosan-modified PLGA (PLGA-CS) membrane was also prepared using a method similar to the preparation of PLGA-HACC [40]. All fibers were dried under vacuum at 37 °C for 24 h.

### 2.4. Characterization

Attenuated total reflectance-Fourier transform infrared (ATR-FTIR) spectroscopy of the membranes was recorded on a spectrophotometer (Perkin-Elmer Co., Waltham, MA, USA) at wavelengths ranging from 800–2400 cm^−1^ and at a resolution of 4.0 cm^−1^ over 16 scans. The morphologies of the electrospun PLGA, PLGA-CS and PLGA-HACC membranes were observed using scanning electron microscopy (SEM, HITACHI SU8220, Tokyo, Japan). The elemental composition of the PLGA-HACC membrane surface was detected using an energy dispersive X-ray spectrometer (EDX) (HITACHI, Tokyo, Japan). The topography of the membranes was also determined by atomic force microscopy (AFM, XE-100, Park SYSTEMS Co., Suwon, Korea). The two-dimensional images were converted to three-dimensional images, and a roughness analysis was done using XEP Data Acquisition Program (Park SYSTEMS Co., Suwon, Korea).

The amount of grafted HACC and chitosan on the surface of the membranes was measured via thermogravimetric analysis (TGA, TA Q-200, New Castle, DE, USA) from 30–700 °C at a rate of 10 °C/min heating with a nitrogen flow rate of 50 mL·min^−1^. The CS and HACC grafting ratio in PLGA-HACC and PLGA-CS was calculated according to the following equation [41]:

Grafting ratio (%) = (*WL*_PLGA_ (%) − *WL*_PLGA-HACC_ (%))/(100% − *WL*_HACC_ (%)) (1)

*WL*: weight loss. For calculation the grafting ratio of CS, *WL*_PLGA-HACC_ and *WL*_HACC_ were changed to *WL*_PLGA-cs_ and *WL*_cs_.

The fiber diameter and pore size were measured by using ImageJ software (National Institutes of Health, Bethesda, MD, USA) on SEM micrographs at 30 random locations. 

The porosity of the membrane was calculated by using Equation (2), respectively [42]:

(2) $$\mathrm{Porosity}=\frac{w_{e}-w_{0}}{\rho_{e}v_{s}}\times 100$$

where ρ_e_ represents the density of ethanol (0.789 g/cm^3^), *V*_s_ represents the geometrical volume (*V*_s_) of the samples, *W*_0_ represents dry samples’ weight and *W*_e_ represents wet samples’ weight. 

### 2.5. Antibacterial Assays

Bacterial activity and morphology on the membranes at 24 h was determined using SEM and confocal laser scanning microscopy (CLSM, Leica TCS SP8, Leica Microsystems, Mannheim, Germany) observation. A volume of 500 μL of the *S. aureus* and *P. aeruginosa* bacterial suspensions in tryptic soy broth (TSB) medium (1 × 10^6^ CFUs/mL) was added into wells containing PLGA, PLGA-CS and PLGA-HACC membranes and then incubated at 37 °C for 24 h. Afterwards, the three samples were gently washed with PBS two to three times to remove loosely-adherent *S. aureus* and *P. aeruginosa* and then sonicated for 5 min with an ultrasonic apparatus to re-suspend the bacteria. After that, the sonicated solution was serially diluted 100-fold using TSB, and 50 μL of the diluted bacteria solution were plated in triplicate onto tryptic soy agar (TSA) plates. Then, after 24 h of incubation in an incubator at 37 °C, the number of bacteria colonies on the plates was counted and multiplied by the dilution ratio. The bacteria growth on membranes was also observed using CLSM. After 24 h of culture, the samples were fixed with 2.5% glutaraldehyde for 30 min at 37 °C. The membranes were then stained in a fresh 24-well plate with 500 μL of combination dye (LIVE/DEAD BacLight viability kits, L7012; Molecular Probes, Life Technologies, Carlsbad, CA, USA). The images were acquired at random positions within the membranes. *S. aureus* and *P. aeruginosa* adhesion on PLGA, PLGA-CS and PLGA-HACC membranes was also observed using SEM. The PLGA, PLGA-CS and PLGA-HACC membranes were incubated with *S. aureus* or *P. aeruginosa* suspended in TSB at a concentration of 1 × 10^6^ CFUs/mL. After incubation for 24 h, the three membranes were fixed in 2.5% glutaraldehyde for 30 min at 37 °C, then dehydrated through a series of graded ethanol solutions (50%, 70%, 95% and 100%). The samples were subsequently dried at 37 °C and then observed using SEM.

### 2.6. Cell Attachment and Proliferation

The three membranes (1 cm × 1 cm) were cultured with HDFs and HaCaTs at a density of 1 × 10^6^ cells/mL in 24-well plates. HDFs’ and HaCaTs’ attachment on the membranes was observed using CLSM after staining with 4,6-diamidino-2-phenylindole (DAPI), and the cell number on membranes (150 μm × 150 μm, *n* = 5) was calculated by ImageJ. The proliferation of HDFs and HaCaTs on the membranes was also tested using the cell counting kit-8 (CCK-8) assay [13]. The absorbance was measured spectrophotometrically at wavelengths of 450 nm with the microplate reader. HDFs and HaCaTs with a density of 2.0 × 10^4^/cm^2^ were seeded on the membrane surface and incubated for 1, 4 and 7 days. The CCK-8 assay was applied to evaluate cell proliferation according to the manufacturer’s instructions.

The morphologies of HDFs and HaCaTs on the membrane surface were also observed using CLSM. The seeding procedures were similar to those of the cell attachment assay. After incubating for 24 h, the cell cultured membranes were fixed with 2.5% glutaraldehyde for 30 min, then stained with rhodamine-labelled phalloidin for 45 min and then stained with DAPI for 10 min. The samples were washed with PBS, and then, CLSM was used to visualize the cell cytoskeleton and the nuclei on the membranes.

### 2.7. HDFs Migration Assay

The dissolution products of the membranes were prepared for the migration assay. One gram of each kind of membrane was soaked in 10 mL DMEM and incubated for 24 h, and the resultant solution was obtained. HDFs were seeded in 24-well plates at 4 × 10^5^ cells per well and cultured in an incubator humidified at 37 °C with 5% CO_2_. After 24 h of culture, a scratch was made with a 200-μL pipette tip at the bottom of each well followed by washing the cells with PBS. The culture medium was then replaced with PLGA, PLGA-CS and PLGA-HACC dissolution products in order to determine the effect of the materials on HDF migration. The cells cultured with DMEM (without FBS) were regarded as a control. After being cultured for 12 h, the cells were fixed with 4% paraformaldehyde for 30 min, and after being washed twice with PBS, the cells were observed and images taken with an inverted microscope (Leica DMI 3000B, Wetzlar, Germany). In addition, a statistical analysis of the HDF migration assay was performed. We measured the original width and final width of the scratches in the two groups, and the percentage of scratch shrinkage was calculated using a previously reported method [43]:

scratch shrinkage (%) = (original width − final width)/original width × 100(3)

### 2.8. Enzyme-Linked Immunosorbent Assay

HDF cells at a density of 1 × 10^6^ cells/mL were incubated with PLGA, PLGA-CS and PLGA-HACC membranes in 24-well plates. After incubation for 24 h, the cell culture medium was collected and centrifuged. The expression levels of FGF-2 were determined via an enzyme-linked immunosorbent assay (ELISA) kit, according to the instructions of the manufacturer [44].

### 2.9. Western Blot Analysis

HDF cells at 2 × 10^6^ cells/well were cultured with PLGA, PLGA-CS and PLGA-HACC in 6-well plates for 24 h. At the 24-h time point, attached HDF cells were washed with PBS, digested with trypsin and dissolved in a lysis buffer containing a protease inhibitor (Roche, Grenzach, Germany). Total protein was isolated from the cell homogenates, subjected to a 10% polyacrylamide gel (Invitrogen, Carlsbad, CA, USA) and transferred to 0.22-μm nitrocellulose membranes (Invitrogen). Mouse anti-human PCNA (Abcam, Cambridge, UK) was used as the primary antibody and incubated with a horseradish peroxidase-conjugated secondary antibody (Santa Cruz Biotechnology, Santa Cruz, CA, USA) for 1 h at 37 °C. The band images were obtained using a ChemiDoc^TM^ XRS + System with Image Lab^TM^ Software (Bio-Rad, Hercules, CA, USA). 

### 2.10. In Vivo Studies Using a Full-Thickness Excision Wound Healing Mice Model

BALB/c mice (four weeks old) were used in the animal study. All animal experimental procedures were performed according to the guidelines of the Animal Ethics Committee of Shanghai Ninth People’s Hospital (No. HKDL 2017100). The mice dorsum skin was shaved and then disinfected using 75% ethanol after anesthetization through the intraperitoneal injection of pentobarbitone sodium at a concentration of 50 mg/kg. An open excision type wound with a diameter of 1 cm was incised on the dorsum of each rat, then a 100-μL *S. aureus* suspension (1 × 10 ^8^ CFU/mL) was injected onto the wound surface and then covered with PLGA, PLGA-CS and PLGA-HACC fibrous membranes, each of which were fixed in place with a bandage. Samples were taken from the center of wound after 3 and 7 days and cultured to evaluate the antibacterial activity. Wound closure observation was assessed on Days 3, 7, 11 and 15 post surgery. This is in accordance with previously reported protocols for such studies [45]. The wound closure rate is expressed by the following equation from a previous study [46]:

Wound size reduction (%) = (*A*_o_ − *A*_t_)/*A*_o_ × 100% (4)

where *A*_o_ is the original wound area and *A*_t_ is the wound area at a specified time point. 

The wound sections with adjacent normal skin were excised for histology and fixed with 10% formaldehyde for the further histological analysis. The tissue samples were then analyzed by H&E and Masson’s trichrome staining for histological observation. 

### 2.11. Statistical Analysis

All data are presented as the mean ± standard deviation (SD). The statistical significance was assessed by analysis of variance (ANOVA). Each result is an average of at least three parallel experiments. 

## 3. Results 

### 3.1. Physical Characteristics

**Figure 1:**
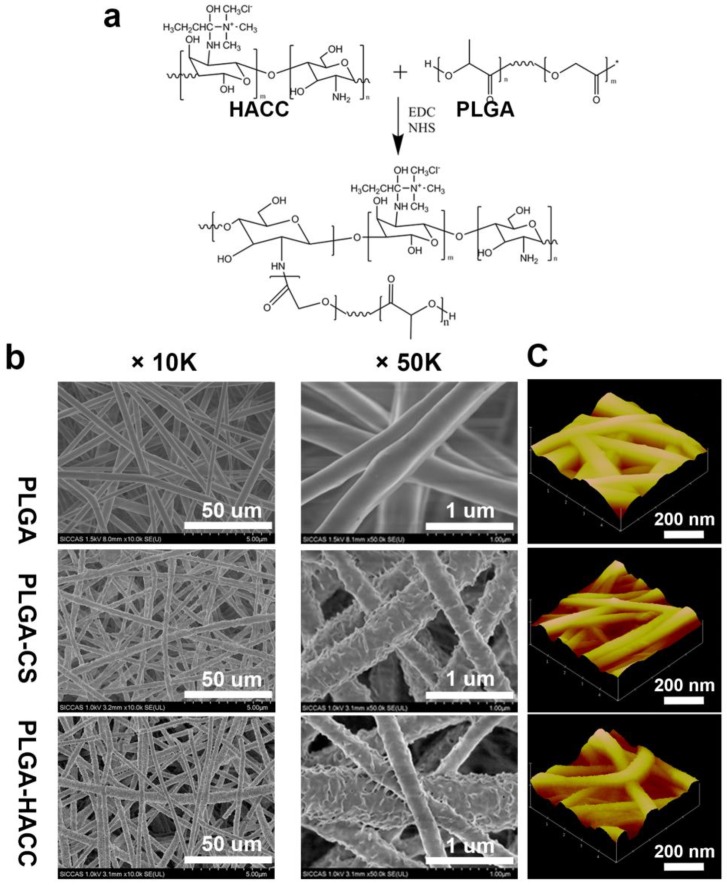
Schematic illustration of HACC-modified electrospun PLGA nanofibrous membrane (**a**) and SEM images (**b**) and AFM images (**c**) of the pure PLGA, the PLGA-chitosan (CS) and PLGA-HACC fibrous membranes.

The HACC-functionalized PLGA membranes were synthesized using electrospinning and surface modification as illustrated in Figure 1a. The carbonyl groups in the PLGA membranes were activated as carboxyl in NaOH solution, then carboxyl groups of PLGA reacted with amido on HACC chains in EDC·HCl/NHS/MES solution. Figure 1b presents SEM images of the PLGA, PLGA-CS and PLGA-HACC fibrous membranes. The electrospun PLGA membranes have a randomly interconnected structure with no formed beads and smooth nanofibers with a diameter of several hundred nano-meters (Table 1). The porosities of the membranes are all in the range of 60–80% and with the pore size in the range of 2.6–3.3 μm; whereas, the surfaces of the PLGA-HACC and PLGA-CS nanofibers appear rough, which is possibly due to the alkali treatment and HACC or CS layer immobilization. More importantly, the HACC and CS surface modification process did not deform the fibrous structure of PLGA, as is clearly evident in the SEM images (Figure 1b). The evolution of the topography of the PLGA, PLGA-CS and PLGA-HACC membranes’ surfaces as observed by AFM is illustrated in Figure 1c. The virgin PLGA film had a relatively smooth surface. The surfaces became rougher with CS and HACC immobilization.

**Figure 2:**
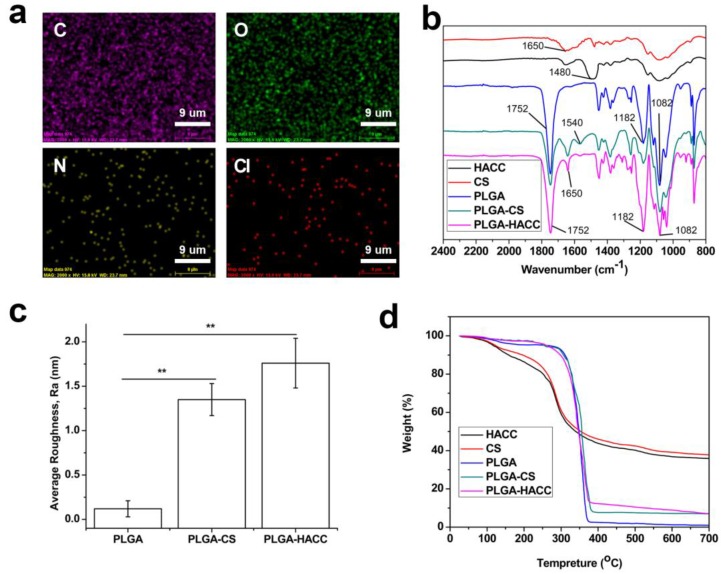
EDX mapping of C, O, N and Cl distribution within PLGA-HACC fibrous membranes (**a**); ATR-FTIR spectra (**b**); average roughness (Ra) of PLGA HACC and PLGA-CS and PLGA-HACC membranes (**c**) and TGA thermogram curves of the CS, PLGA, HACC and PLGA-CS and PLGA-HACC fibrous membranes (**d**); ** indicates *p* < 0.05.

Figure 2a shows the C, O, N and Cl elemental distributions in the PLGA-HACC membranes as detected by EDX mapping, and the elemental N derived from HACC in the membranes is illustrated. The ATR-FTIR spectra of PLGA, PLGA-HACC and the PLGA-CS membranes are shown in Figure 2b. It can be clearly observed that pure PLGA had a peak at 1752 cm^−1^ (carbonyl –C=O stretch), as well as peaks at 1182 and 1082 cm^−1^ (C–O–C ether group) [47]. The characteristic peak at 1650 cm^−^^1^ represents amide Ι, and 1540 cm^−^^1^ corresponds to amide II in CS. For the HACC, the peak at 1480 cm^−^^1^ was assigned to the C–H bending of the trimethylammonium group [33]. The FTIR spectra of the PLGA-CS and PLGA-HACC membranes exhibited peaks at 1650 and 1540 cm^−^^1^, suggesting that CS and HACC had been successfully immobilized on the PLGA membranes. The PLGA membranes showed an average roughness of 0.12 ± 0.09 nm. The CS and HACC immobilization led to increased average roughness of 1.35 ± 0.18 and 1.76 ± 0.28 nm (Figure 2b). 

The thermal characteristics of the PLGA membrane samples before and after CS or HACC surface modification were investigated using TGA (Figure 2d). The PLGA only membranes started to degrade at about 300 °C, and degradation finished at around 380 °C with complete weight loss occurring. The PLGA-HACC and PLGA-CS membranes had the same behavior in the starting degradation temperatures. The thermal degradation of pure CS and HACC started at 220 °C, and 64.08% and 64.29% weight, respectively, was lost at about 700 °C. The membranes were heated up to 700 °C, and at that temperature, the PLGA completely degraded, with only CS or HACC left. The total weight losses were 92.36% and 93.02% for the PLGA-HACC and PLGA-CS membranes, respectively. The grafting ratios of the PLGA-CS and PLGA-HACC membranes were 21.39% and 19.43%. 

### 3.2. Attachment, Spreading and Proliferation of HDFs and HaCaTs

**Figure 3:**
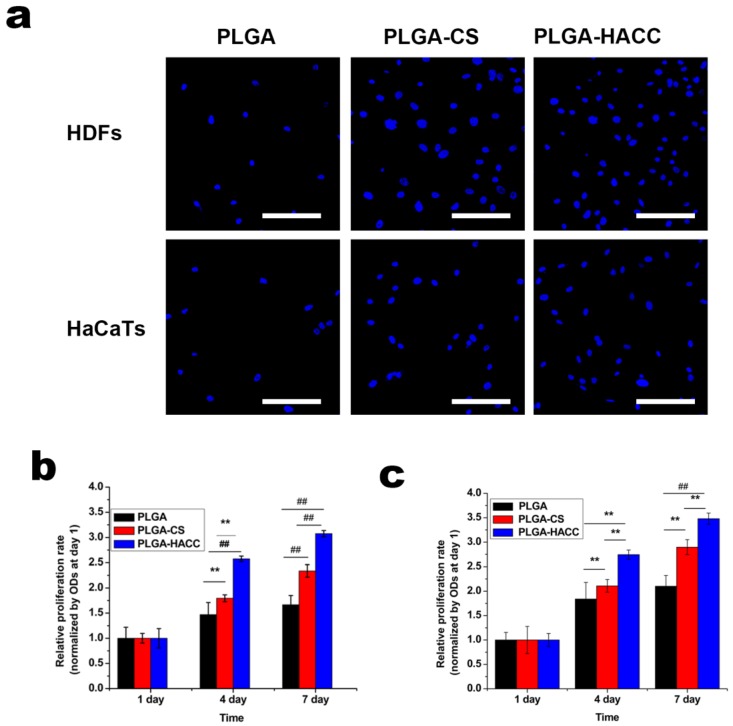
Attachment and proliferation of human dermal fibroblasts (HDFs) and HaCaTs on various fibrous membrane surfaces. Cells stained with DAPI after 6 h of culture (**a**); relative HDFs’ (**b**) and HaCaTs’ (**c**) proliferation rates on various fibrous membranes as measured by the CCK-8 assay; ## indicates *p* < 0.01, and ** indicates *p* < 0.05. The scale bar is 50 μm.

DAPI staining was used to evaluate cell attachment. Figure 3a shows the numbers of HDFs and HaCaTs on the surfaces of the three different membranes after 6 h of culture stained with DAPI. The cell number on the surface of membranes was calculated by ImageJ. The numbers of adherent HDFs and HaCaTs on the surfaces of the PLGA-CS (63 ± 7, 35 ± 8) and PLGA-HACC (82 ± 9, 52 ± 8) membranes were significantly higher than those on the PLGA membranes (18 ± 5, 16 ± 7). More HDFs and HaCaTs attached to PLGA-HACC than to PLGA-CS at the 6-h time point (Figure 3a).

The proliferation rates of HDFs and HaCaTs cultured on membrane surfaces is shown in Figure 3b,c. The proliferation rates of HDFs on PLGA are not very promising from Day 1–Day 7. HDF cells on the PLGA-HACC membranes showed a higher proliferation rate compared with those on the PLGA and PLGA-CS membranes at Day 4 and Day 7. The HDFs numbers on the PLGA-HACC membranes increased significantly from Day 1–Day 7. The same trend is also observed in the case of HaCaTs (Figure 3c). A significantly higher growth rate of the proliferation rates of the HDFs and HaCaTs on the PLGA-HACC was observed compared to that on PLGA and PLGA-CS.

### 3.3. Cellular Morphology

**Figure 4:**
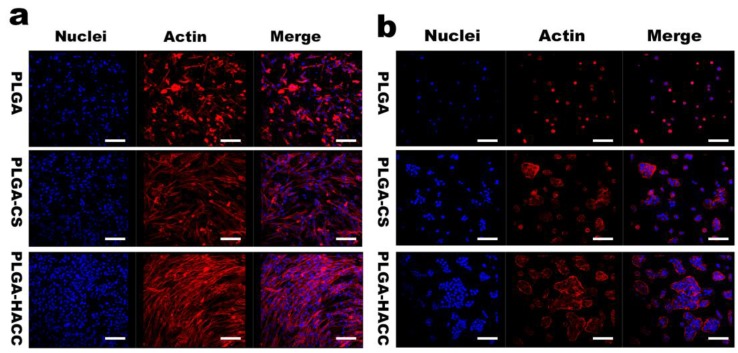
The cytoskeletal morphology of HDFs (**a**) and HaCaTs (**b**) on different fibrous membrane surfaces after incubation for 24 h. Representative images of cells stained with rhodamine-labeled phalloidin for actin filaments (red) and DAPI for nuclei (blue). The scale bar is 50 μm.

Figure 4 shows the spreading of HDFs and HaCaTs cells on PLGA, PLGA-CS and PLGA-HACC membranes’ surfaces as observed by CLSM. As shown in the CLSM micrographs, after 24 h of incubation, the cells grown on the PLGA-HACC membranes displayed more actin filaments linking adjacent cells and had a characteristic shape; however, the cells on the PLGA membranes exhibited poor spreading and had a dispersed monolayer with fewer actin filaments. The cell density and morphology were better for the PLGA-CS membranes than for the PLGA membranes. 

### 3.4. Enhanced Regenerative Activities of Skin Cells Cultured on PLGA-HACC In Vitro

**Figure 5:**
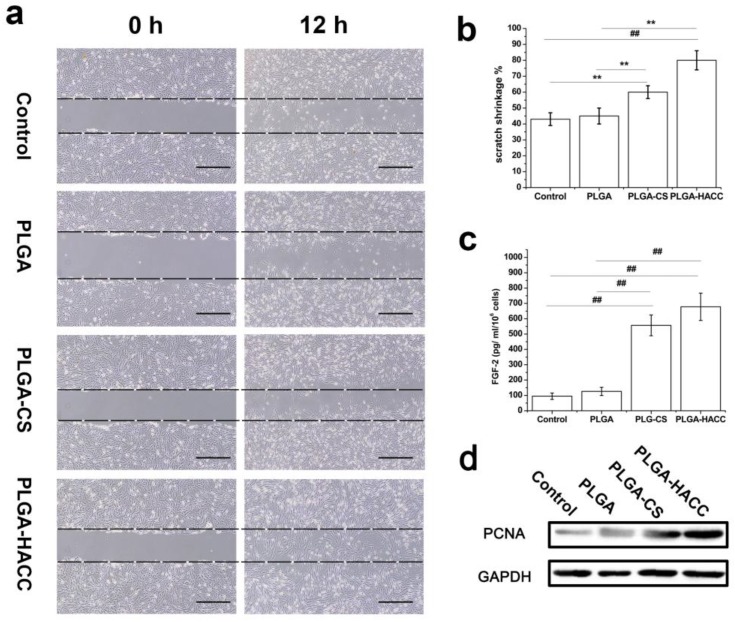
Migration of HDFs cultured with different fibrous membrane extract solution (**a**); statistical analysis of HDFs migrations (**b**); FGF-2 production in HDFs 24 h after treatment as determined by ELISA (*n* = 5) (**c**); Western blotting of PCNA expression in dermal fibroblast proliferation (**d**); ## indicates *p* < 0.01, and ** indicates *p* < 0.05. The scale bar is 100 μm.

HDF is believed to play a key role in wound healing by synthesizing extracellular matrix components, which allow the epithelial cells of to affix to the matrix, thereby allowing the epidermal cells to effectively join together to form the top layer of the skin. Cell migration is a complex multistep process that involves the movement of cells from one area to another and plays a vital role in wound repair. The wound scratch model is a 2D assay for evaluating wound healing in vitro [48]. The membrane dissolution products with control medium (without FBS) were used to detect the effects of membranes on HDF migration and the results are shown in Figure 5a. At 0 h, scratches with the same width were made on the bottom of each well covered with HDFs. Next, the cells were cultured with control medium and PLGA, PLGA-CS and PLGA-HACC dissolution products for 12 h: The scratches in the control, PLGA and PLGA-CS groups became slightly narrower, while the scratch in the PLGA-HACC-containing group almost disappeared, which indicates that HACC stimulated the HDFs to migrate into the scratch area. Figure 5b shows the statistical analysis of the HDF migration assay; the scratch shrinkage percentage in the PLGA-HACC group (83 ± 3.5%) was much higher than that in the PLGA-CS (58.3 ± 4.2%), PLGA (44 ± 3.9%) and control groups (42 ± 3.5%).

To evaluate the effects of PLGA-HACC on skin cells in vitro, HDFs were cultured for 24 h with PLGA, PLGA-CS and PLGA-HACC membranes. Fibroblast growth factor (FGF-2) production from HDFs was enhanced in the PLGA-HACC group (Figure 5c). Compared with the cells cultured on PLGA, dermal fibroblasts cultured with PLGA-HACC showed an increase in cell proliferation (expression of proliferating cell nucleus antigen (PCNA) (Figure 5d). This is particularly important because wound healing requires fibroblast proliferation and differentiation into myofibroblasts [43,49]. The above results suggested that the PLGA-HACC could accelerate wound healing. 

### 3.5. Antibacterial Efficacy of Different Membranes

**Figure 6:**
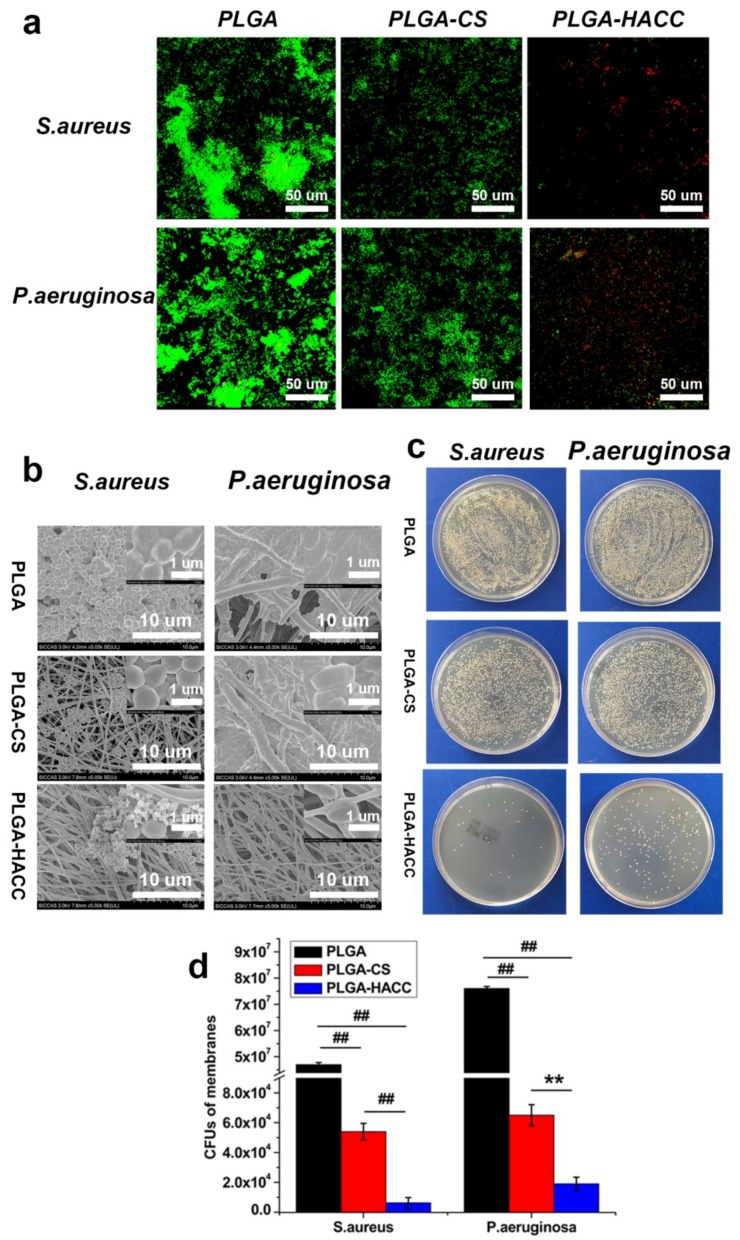
Images of *S. aureus* and *P. aeruginosa* activity on the surfaces of PLGA, PLGA-CS and PLGA-HACC fibrous membranes at 24 h obtained using CLSM (**a**); SEM images of *S. aureus* and *P. aeruginosa* incubation with PLGA, PLGA-CS and PLGA-HACC fibrous membranes at 24 h; the inset shows SEM images at a higher magnification (**b**); photographs of bacterial colonies formed by *S. aureus* and *P. aeruginosa* after incubation with PLGA, PLGA-CS and PLGA-HACC fibrous membranes for 24 h (**c**); quantity of adherent bacteria on the three membranes after 24 h of incubation (**d**); ## indicates *p* < 0.01, and ** indicates *p* < 0.05.

The bacterial activities of *P. aeruginosa* and *S. aureus* on the PLGA, PLGA-CS and PLGA-HACC membranes at the 24 h time point were observed using both SEM and CLSM. Considerably less live bacteria (appearing as green fluorescence) could be observed on the PLGA-HACC membranes compared to the PLGA and PLGA-CS membranes, which indicates significantly less adherent surviving bacteria on the PLGA-HACC than on the PLGA membranes. A considerably density of dead bacteria (appearing as red fluorescence) indicated that dead colonies could be observed on the PLGA-HACC membranes. Figure 6b shows SEM images of bacterial morphology on the PLGA, PLGA-CS and PLGA-HACC membranes. *S. aureus* and *P. aeruginosa* showed more attachment on PLGA membranes compared to the PLGA-CS and PLGA-HACC membranes. A decrease in bacterial attachment was also seen in PLGA-CS membranes compared with the PLGA membranes. Very few sparsely-distributed *S. aureus* and *P. aeruginosa* could be spotted over the entire surface of the PLGA-HACC fibrous membranes. The bacteria obviously exhibit impaired structure, and cell membranes began to fester, which indicated that these bacteria were inactivated on the PLGA-HACC surface. Figure 6c,d quantitatively shows the surviving *S. aureus* and *P. aeruginosa* strains on the membranes at 24 h as determined by the spreading plate method.

The numbers of *S. aureus* and *P. aeruginosa* on PLGA-HACC membrane surfaces were found to be significantly less than those on the PLGA and PLGA-CS membrane surfaces. It is evident that there are less viable bacteria on PLGA-CS than that on the surface of PLGA. The *S. aureus* and *P. aeruginosa* burden per membrane of the PLGA (4.78 × 10^7^ ± 7.8 × 10^5^, 7.60 × 10^7^ ± 8.45 × 10^5^ CFUs/membrane, respectively) and PLGA-CS (5.43 × 10^4^ ± 5.50 × 10^3^, 6.53 × 10^4^ ± 7.14 × 10^3^ CFUs/membrane, respectively) was significantly higher than that of the PLGA-HACC (6.31 × 10^3^ ± 3.40 × 10^3^, 1.92 × 10^4^ ± 4.48 × 10^3^ CFUs/membrane, respectively) after 24 h of incubation (Figure 6d). Thus, HACC demonstrated very effective antibacterial activity against *S. aureus* and *P. aeruginosa*. 

### 3.6. In Vivo Wound Healing

**Figure 7:**
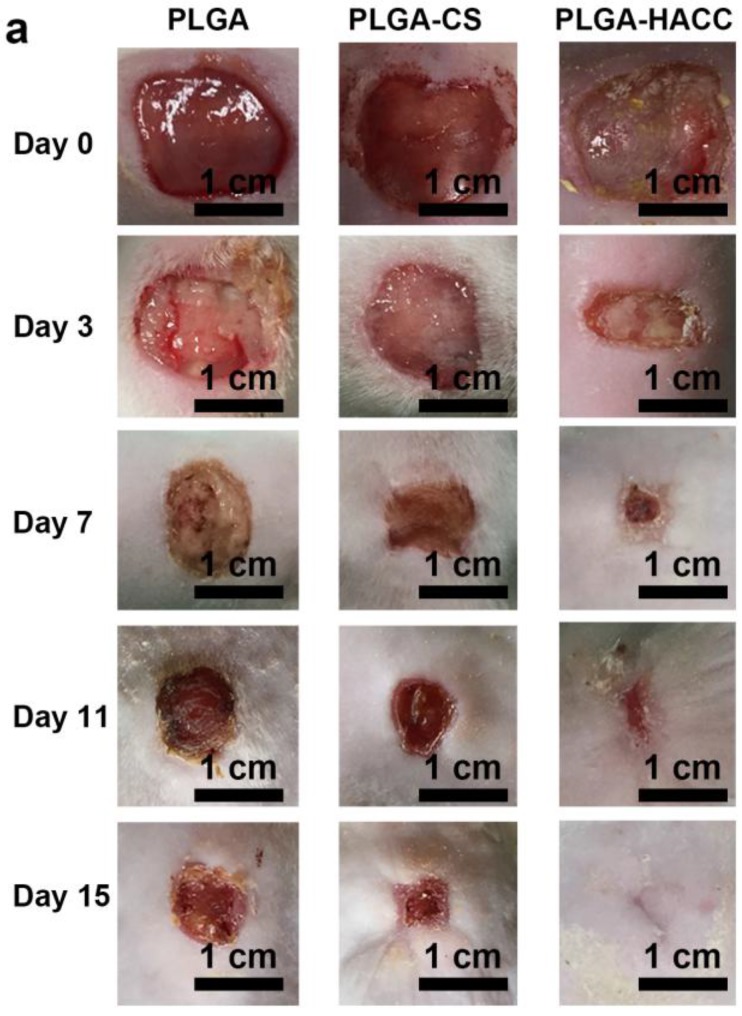

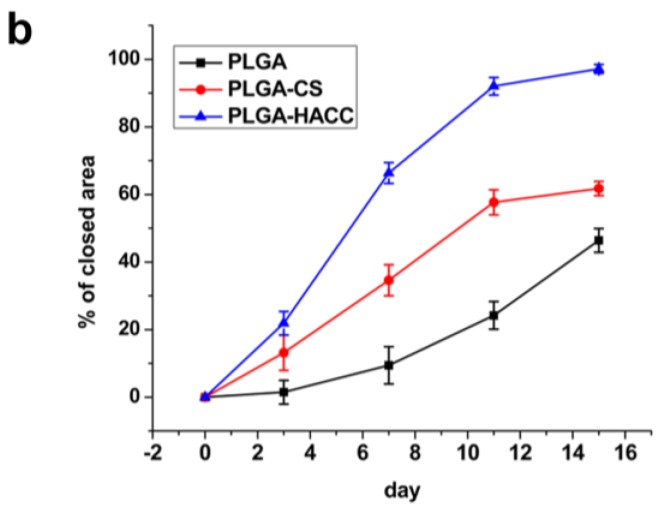
Representative photographs of the *S. aureus*-infected wound untreated and treated with PLGA, PLGA-CS and PLGA-HACC fibrous membranes after 3, 7, 11 and 15 days (**a**) and their corresponding (quantitatively measured) wound size reduction (**b**).

We used a full-thickness infected cutaneous wound model to evaluate the healing characteristics of PLGA-HACC membranes in vivo. Figure 7a shows optical microscopic images of wound cuts treated with PLGA, PLGA-CS and PLGA-HACC fibrous membranes for 3, 7, 11 and 15 days. Differing from the PLGA and PLGA-CS groups, PLGA-HACC reduces the wound size by 21.8% after three days (Figure 7a,b). After seven days of treatment, the wound size of the PLGA membrane group was not significantly reduced, while both PLGA-CS and PLGA-HACC were able to reduce the wound size, by 34.5% and 66.4%, respectively (Figure 7b). After 11 days of treatment with either PLGA, PLGA-CS or PLGA-HACC, the wound size was reduced by 24.2%, 57.1% and 91.8%, respectively (Figure 7b). After 15 days of treatment, PLGA-HACC had a wound healing ratio of nearly 100%, significantly higher than that of the PLGA (46.4%) and PLGA-CS groups (61.3%) (Figure 7b). Such a strong wound-healing effect of PLGA-HACC could be attributed to the synergistic effects between antibacterial performance and cell migration promotion by HACC. 

**Figure 8:**
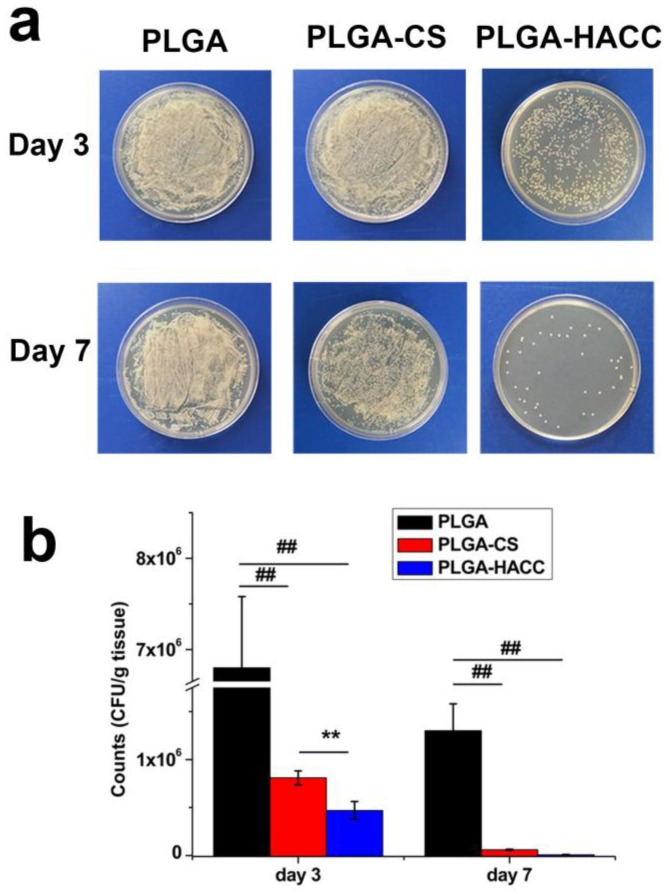
Bacteria separated from wound tissue were cultured on agar plates at Day 3 and Day 7 (**a**); The number of the surviving bacteria in the wound tissue of each sample (**b**); ## indicates *p* < 0.01, and ** indicates *p* < 0.05.

To determine the amounts of bacteria in infected tissue samples, the tissue was homogenized in normal saline (1.0 mL) and plated on LB agar, and the number of colonies was counted. Homogenized tissue dispersions from the infection site were added to bacteria-coated plates to evaluate therapeutic efficacy (Figure 8). *S. aureus* load in the infected tissue at Day 3 was 6.6 × 10^6^ CFU·g^−1^ in mice treated with PLGA and was reduced by PLGA-CS and PLGA-HACC treatment to 8.1 × 10^5^ and 2.1 × 10^4^, respectively. At Day 7, the bacterial load in infected tissue mice was 1.3 × 10^6^, and 6.1 × 10^4^ CFU·g^−1^ in response to PLGA and PLGA-HACC treatment, respectively, while the PLGA-HACC treatment reduced the bacterial load to 3.2 × 10^3^ CFU·g^−1^. 

**Figure 9:**
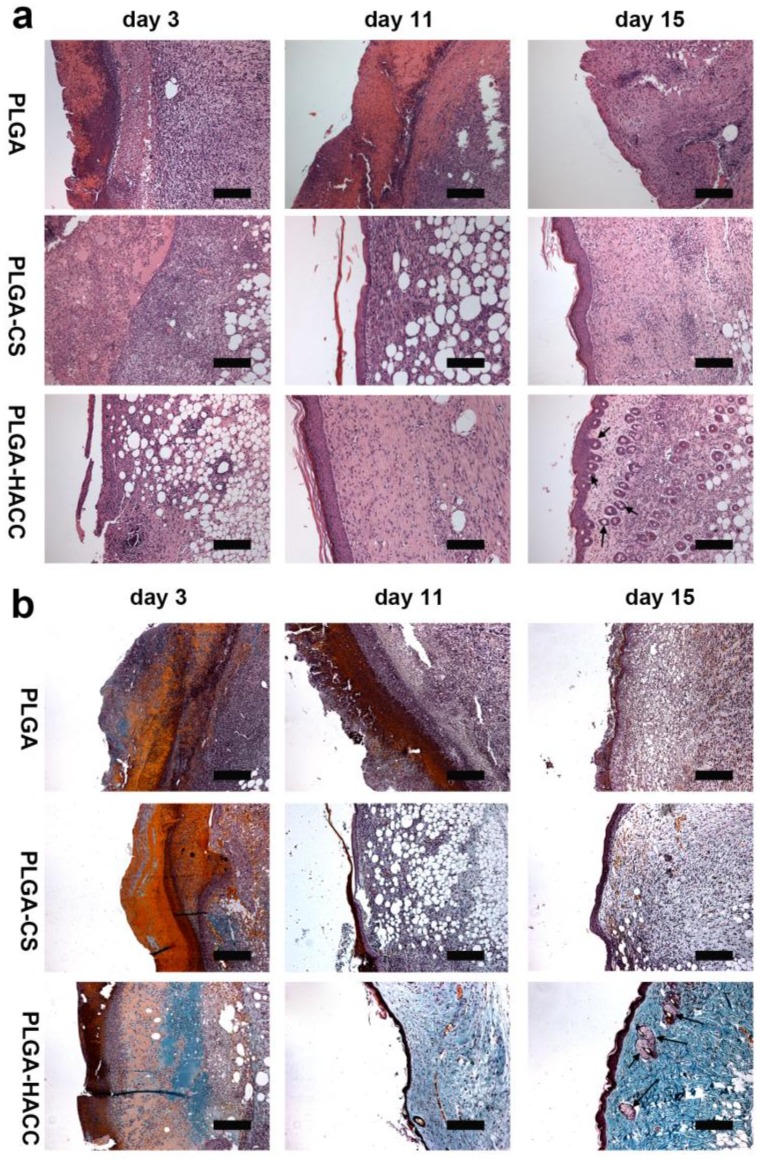
HE staining (**a**) and Masson’s trichrome staining (**b**) of wound healing in the groups of PLGA, PLGA-CS and PLGA-HACC fibrous membranes. The scale bar is 50 μm.

Histomorphological determination of wound regeneration at different phases was conducted by HE and Masson’s trichrome staining (Figure 9). At Day 3, there was enhanced infiltration of inflammatory cells in both the PLGA and PLGA-CS dressing groups, especially for macrophages and other monocytes in the PLGA group. Compared with the PLGA and PLGA-CS groups, inflammatory cell infiltration was partially suppressed, and far more fibroblasts were gathered around the impaired region for PLGA-HACC. On Day 11, many inflammatory cells appeared on the PLGA-treated wound. The PLGA-CS group’s wounds showed a faster healing rate than the PLGA group. Compared with the PLGA and PLGA-CS groups, the PLGA-HACC group showed a higher regularity of both epithelium and connective tissue with more fibroblasts and a more complete epithelial structure than the two control groups, which might be attributed to the high antibacterial efficiency and the promotion of cellular activities (such as proliferation and migration), including the activities of fibroblasts and keratinocytes, by HACC. After 15 days of treatment, many inflammatory cells still appeared on the PLGA-treated wounds, compared with the wounds treated with PLGA-CS membranes. In addition, after 15 of days treatment, PLGA-HACC dressings led to the production of hair follicles (indicated by black arrows). Inflammatory cells disappeared from the PLGA-HACC-treated wounds after 15 days, and the PLGA-treated wounds were covered by an incomplete epidermis. A thickened and complete epidermis was observed in the PLGA-HACC membrane groups.

Masson’s Trichrome staining (Figure 9b) was performed to assess the collagen deposition (seen as blue) in the wound site. At both Day 3 and 11 of treatment, the PLGA-HACC treatment groups showed significantly higher collagen deposition than the PLGA and PLGA-CS groups. After 15 days of treatment, compared to the PLGA and PLGA-CS treatment groups, more mature collagen fibers and the production of hair follicles (indicated by black arrows) were also observed in the PLGA-HACC treatment group with less inflammatory cell presence, which is consistent with the HE staining results. More collagen tissue could help in the reconstruction of the ECM and further support skin tissue growth.

## 4. Discussion

The aim of the current study was to fabricate PLGA electrospun membranes with HACC surface modification to repair infected wounds. The SEM and AFM images showed that the prepared PLGA-HACC membranes continue to have a uniform, porous structure after the immobilization process (Figure 1). The nanoscale topography of the PLGA-HACC membranes mimics the natural extracellular matrix and is, thus, favorable for cell attachment migration and proliferation [50]. The porosity of the prepared membranes favors nutrient and gas exchange in wound treatment, which is beneficial for wound healing [51]. 

During wound healing, infections caused by pathogens delay the closing of the wound and increase the healthcare burden [52,53]. Among a plethora of different antiseptics for wound care (mostly alcohols, hydrogen peroxide and iodine), there have already been attempts to load silver into electrospun nanofibers [54]. Because the utilization of antimicrobial agents can cause toxicity and the development of drug resistance, other approaches are highly desired. Our previous study found that HACC with moderate DS was not cytotoxic and had high antibacterial activity. In this study, HACC with 30% DS was grafted onto PLGA nanofibers to prevent post-wound infections. Based on the in vitro (Figure 6) results, PLGA-HACC exhibited effective antibacterial activity towards both Gram-positive (*S. aureus*) and Gram-negative (*P. aeruginosa*) bacteria. Our previous study demonstrated that HACC exhibited a broad spectrum of antibacterial ability against various Gram-positive bacteria, which may be due to the electrostatic interaction of the positively-charged HACC and the negatively-charged bacterial membranes, which causes cytoplasmic membrane fracture of bacteria cells [34]. In addition, it can also be expected that the application of this material will not induce bacterial resistance. 

PLGA is a synthetic polymer that is used as a biomedical material because of its good biocompatibility, adjustable mechanical properties and tunable degradation rate [41]. However, the electrospun PLGA membranes had a relatively smooth surface (Figure 1) and support low levels of cell adhesion (Figure 3). The surfaces became rougher after alkali treatment and CS and HACC immobilization. More cells adhered to the surface of the PLGA-HACC membranes, and roughness of the PLGA-HACC surface might have contributed to these results. Several other studies have shown that cell response is improved by rough material surfaces [55,56]. 

It was demonstrated that the PLGA-HACC membranes could promote the migration of HDFs with increased PCNA and FGF-2 expression (Figure 5), which enhances myofibroblastic differentiation, the growth and migration of dermal fibroblasts, angiogenesis and wound healing [49]. We concluded that the material made by grafting HACC onto PLGA membrane surfaces demonstrated acceptable cytocompatibility, as well as good antimicrobial activity. The in vivo experiments furtherly demonstrated that the PLGA-HACC membranes were effective in reducing the inflammatory response after implantation into the infected wound (Figure 9). As expected, type I collagen was the most expressed form of collagen in the skin, serving as the framework for connecting skin tissue [57]. The PLGA-HACC membranes stimulated COL expression on Day 11 and Day 15 in infected wound skin, and using these membranes also resulted in higher collagen production, as observed by Masson’s trichrome staining (Figure 9b).

## 5. Conclusions

In this study, HACC-modified PLGA nanofibrous membranes were fabricated through entrapment-graft treatment. Bacteria colonization on the surface of PLGA-HACC was observed using SEM and CLSM. Compared with PLGA and PLGA-CS membranes, PLGA-HACC membranes exhibited effective antibacterial activity towards both Gram-positive (*S. aureus*) and Gram-negative (*P. aeruginosa*) bacteria. HACC modification exhibited favorable cytocompatibility and significant ability to stimulate the adhesion, spread and proliferation of HDFs and HaCaTs. The in vivo study demonstrated that PLGA-HACC exhibits excellent wound healing efficacy in an infected full-thickness excision wound model in mice, which showed significant re-epithelialization and dermal reconstruction.

## Figures and Tables

**Table 1:** Fiber diameter, pore size, porosity and average roughness of PLGA, PLGA-CS and PLGA-HACC electrospun membranes.

Sample	Fiber Diameter (nm)	Pore Size (um)	Porosity (%)	Average Roughness (nm)
PLGA	325.9 ± 32.5	3.3 ± 0.5	71.5 ± 5.6	0.12 ± 0.09
PLGA-CS	421.3 ± 38.5	2.8 ± 0.7	66.8 ± 8.5	1.35 ± 0.18
PLGA-HACC	480.1 ± 41.5	2.6 ± 0.6	62.9 ± 5.9	1.76 ± 0.28
